# Generic SDE and GA-based workload modeling for cloud systems

**DOI:** 10.1186/s13677-020-00223-5

**Published:** 2021-01-18

**Authors:** Cédric St-Onge, Souhila Benmakrelouf, Nadjia Kara, Hanine Tout, Claes Edstrom, Rafi Rabipour

**Affiliations:** 1 ÉTS, University of Quebec, Montreal, Canada; 2 Ericsson Canada, Montreal, Canada

**Keywords:** Cloud computing, Workload modeling, Workload estimation, Hull-white model, Genetic algorithm, Support vector regression, Kalman filter

## Abstract

Workload models are typically built based on user and application behavior in a system, limiting them to specific domains. Undoubtedly, such a practice creates a dilemma in a cloud computing (cloud) environment, where a wide range of heterogeneous applications are running and many users have access to these resources. The workload model in such an infrastructure must adapt to the evolution of the system configuration parameters, such as job load fluctuation. The aim of this work is to propose an approach that generates generic workload models (1) which are independent of user behavior and the applications running in the system, and can fit any workload domain and type, (2) model sharp workload variations that are most likely to appear in cloud environments, and (3) with high degree of fidelity with respect to observed data, within a short execution time. We propose two approaches for workload estimation, the first being a Hull-White and Genetic Algorithm (GA) combination, while the second is a Support Vector Regression (SVR) and Kalman-filter combination. Thorough experiments are conducted on real CPU and throughput datasets from virtualized IP Multimedia Subsystem (IMS), Web and cloud environments to study the efficiency of both propositions. The results show a higher accuracy for the Hull-White-GA approach with marginal overhead over the SVR-Kalman-Filter combination.

## Introduction

W ITH the growing ubiquity of cloud computing technologies over the past decade, cloud providers and researchers have strived to design tools for evaluating and enhancing different Quality of Service (QoS) aspects of their systems, mainly performance, availability, reliability and power efficiency. Failing to optimize such aspects can compromise service availability and lead to Service Level Agreement (SLA) violations, and thus, incurring penalties to cloud providers. The development of system management policies that support QoS is therefore crucial. However, the latter is quite challenging, as it must rely on evaluation tools which are capable of accurately representing the behavior of multiple attributes (e.g., CPU, RAM, throughput, network traffic) of cloud systems [1]. It is also complicated by the very essence of cloud systems, which are built on heterogeneous physical infrastructures and experience varying demand. These systems have different physical resources and network configurations and different software stacks. Further, reproducing conditions under which system management policies are evaluated and controlling evaluation conditions are challenging tasks [2].

In this context, workload modeling facilitates performance evaluation and simulation since we can generate at will synthetic workload resource profiles by using a “black box” system. Workload models allow cloud providers to evaluate and simulate resource management policies aimed at enhancing their system QoS before they are deployed in full-scale production environments. For researchers, it provides a controlled input, allowing workload adjustments to fit particular situations, as well as the repetition of evaluation conditions and the inclusion of additional features [1]. In other cases, researchers skilled in the field of Deep Learning (DL) are ofter seeking large quantities of synthetic workload data to train models that can enhance scaling and resource adaptation of Virtual Machines (VMs) in cloud environments such as Network Function Virtualization Infrastructures (NFVIs). Furthermore, workload simulation based on realistic scenarios enables the generation of tracelogs, which are scarce in cloud environments due to commercial and confidentiality concerns. Workload modeling and workload generation are challenging, especially in cloud systems, due to multiple factors: (i) workloads are composed of various tasks and events submitted at any time, (ii) heterogeneous hardware in a cloud infrastructure impacts task execution time and arrival time, and (iii) the virtualization layer of the cloud infrastructure incurs overhead due to additional I/O processing and communications with the Virtual Machine Monitor (VMM). These factors make it difficult to design such models and generators fitting different workload types and attributes. In the current state of the art, effort is instead deployed to design specialized workload modeling techniques focusing mainly on specific user profiles and application types [1, 3–6].

To tackle the above issues, we propose in this work a hybrid workload modeling and optimization approach to accurately estimate CPU and throughput workload, applicable to different domains. The objective is to develop realistic CPU and throughput workload profiles for different virtualized telecom and IT systems, based on predictable workload data obtained from real systems. These workload types differ significantly from each other. For instance, IT (i.e., virtualized Web server) CPU workloads show sharper variations in short bursts while telecom (i.e., virtualized IMS infrastructure) workloads, on the other hand, show flatter, continuous loads under normal customer demand. This is due to the way the CPUs handle the different jobs and tasks from each workload domain, as Web service CPU loads involve distributed computing, while IMS CPU loads generally comprise individual call setup activity. Under critical, unexpected customer demand, however, IMS CPU loads will have dramatic variations, and can impact the whole system as customer calls may be dropped. Hence, our proposition consists first in modeling CPU and throughput workload data sets by using different Hull-White modeling processes, and then determining an optimal estimated workload solution based on a custom Genetic Algorithm (GA) [7].

In this work, we also propose a Kalman filter [8] and support vector regression (SVR) [9] combination to estimate CPU and throughput workloads. First, IMS CPU and throughput observed workloads are used for modeling purposes. They include two CPU load profiles, where CPU workload is generated by stressing a virtualized IMS environment with varying amounts of calls per second, thus producing sharp increases and decreases in CPU workload over long time periods. An IMS throughput load profile is also provided, where throughput variations follow a more steady pattern. Next, the IT workload type includes two different profiles. The Google CPU workload under evaluation, for example, is composed of sharp spikes of CPU workload variations over short periods of time. By contrast, another CPU workload, namely BitBrains, is characterized by very narrow spikes. These datasets therefore display unique trends and behaviors, which provide interesting scenarios to evaluate the efficiency of the workload modeling and workload generation techniques that we are proposing in this paper. The evaluation of the mean absolute percentage error (MAPE) of the best estimated data provided by the proposed Hull-White-based approach against the observed data shows significant improvement in the accuracy level, as compared to other workload modeling approaches, such as the SVR and the SVR with a Kalman filter.

The main contributions of this work consist of:

- Proposing a generic CPU workload modeling approach fitting different workload domains (e.g., telecom, IT).
- Providing an automated workload-generating tool capable of generating an estimated workload with minimal user input.
- Generating workload models without requiring knowledge of the inner behaviors of the modeled systems.
- Generating workload profile data while limiting dependence on external organizations for providing such data.

To achieve the main objective of this work, we use workload modeling for a special case of performance evaluation, namely, capacity planning. In a sense, capacity planning is performance evaluation in reverse. In other words, instead of deriving the performance of a given system configuration, we seek the configuration that will provide the desired performance [10].

### Background

With the rise of cloud computing over the past decade, there has been an increasing amount of research conducted to help cloud providers improve their system performance, through metrics such as energy efficiency and QoS. To achieve this goal, a common practice is to evaluate a system’s workload. While some approaches already exist to tackle online non-predictable workload [11–13], ours focuses on offline predictable workload estimation. The notion of predictable workload denotes a “normal” and continuous usage of system resources. A non-predictable workload, on the other hand, is one which is triggered by a short, unexpected and extreme event. For example, this could be a surge in calls being processed by an IMS system during an earthquake. In the context of cloud computing, this is an important factor to consider since it has an impact on how to handle resource allocation. In [14], the authors propose different methodologies and allocation decisions based on both workload types. In the presence of a predictable workload, for example, their algorithm is designed to invoke a virtual machine (VM) migration only if a fixed threshold is violated for a sustained period of time. In the presence of a non-predictable workload, on the other hand, VM migration may take place when utilization is above a fixed threshold value or, for the case of public clouds for instance, VM scaling may not be automatic; instead, it is common to horizontally scale an application across multiple VMs and perform load balancing. VM migration and vertical scaling may be a manual process initiated by the user. The use of predictable workload data is therefore preferred for building offline workload models where a user wishes to forecast future load, while non-predictable workload data is preferred for building online workload models in which there are variations such as sharp, critical spikes in the load, which might impact the system.

### Motivation

Precisely predicting CPU, memory, network and I/O resource utilization helps cloud providers to meet QoS requirements without breaching SLAs to their customers or their own service level objectives (SLO) by anticipating potential resource provisioning demand in their infrastructure. For instance, the authors in [15, 16] propose novel algorithms to increase power efficiency in datacenter infrastructures while satisfying QoS requirements in intra and inter datacenter networks.

However, authentic industrial-grade workload data is sparse and often incomplete. Also, actual workload models are limited to very specific domains, and are not flexible enough, to build a generalized representation for different domains, such as IT (i.e., Web servers) and telecom (i.e., IMS architecture) virtualized cloud environments. Further, the workload model must adapt to evolution in the system configuration parameters (e.g., load, switched off CPU core). In this context, our proposed approach differs from other existing solutions in various aspects:

- Combining Hull-White processes with a Genetic Algorithm (GA) automates the selection of segmented potential solutions through different models.
- GA reinforces the Hull-White process, and optimizes the workload models by selecting the fittest solutions through many generations.
- Significant improvement in execution time compared to other types of workload estimation models (e.g. auto-regression, moving average).
- Accommodation of on-demand workload profile changes in a simulation thanks to adaptable and continuous spline functions.

To summarize, the Hull-White-GA approach was designed to generate large quantities of synthetic workload data with specific load profiles in mind (aimed at predicting optimal scaling/migration accuracy, efficiency in NFVIs). This data was in turn used as input training data for novel deep learning approaches yet to be published by our team. In this regards, focusing on specific metrics for specific Virtualized Network Functions (VNFs) was our main objective and Hull-White-GA successfully filled this objective.

### Problem illustration

One of the many challenges that arise for researchers attempting to evaluate cloud system policies is the ability to get reliable data and tracelogs from organizations hosting cloud environments. Since getting workload data requires access to an expensive, full-scale deployment of a cloud infrastructure, researchers rely on the willingness of organizations to provide, somewhat reluctantly, such data and tracelogs. To circumvent this challenge, workload modeling is introduced as a good alternative, enabling research teams to generate large amounts of workload profile data for use in the course of their work, while limiting dependence on external organizations to provide such data. Another aspect not covered by existing approaches is to give the ability to researchers to quickly get access to massive quantities of synthetic workload data with specific load profiles. This challenge is especially dire of consequence in areas attempting to leverage DL models for cloud resource adaptation and allocation, where access to input training data is scarce, limiting the efficiency or the designed approaches.

Moreover, current workload modeling solutions rely on probability distribution functions and statistical analysis to model the impact of jobs, tasks and/or applications on a system’s resource utilization. For our part however, the present paper aims to build deterministic prediction models based on a stochastic differential equation (SDE) known as the Hull-White process. This approach differs from others currently found in the literature, as it aims to model the behavior of a system’s resource usage over time, stressed by a known number of tasks and/or jobs, thus creating load profiles. It also enables our models to adapt to the evolution in system configuration parameters (e.g., booting up a new VM or enabling new CPU cores to handle excessive levels of resource loads). The resulting estimated workload values can then be processed through an optimization algorithm, known as a GA, to improve the fidelity of the estimated values as compared to the observed values.

This novel approach thus answers the need not currently filled by other approaches to get a general purpose, generic modeling algorithm that can fit not only different types of workloads, such as virtualized telecom and IT systems’ CPU utilization with a high degree of fidelity to the observed workload, but also generate workload patterns and seasonality commonly found in heterogeneous cloud infrastructures that run distributed applications across multiple VMs and servers. Moreover, this workload-modeling algorithm allows researchers to generate workload traffic and resource usage models:

1. without having to know the specifics of user behavior and the deployed applications;
2. with a high degree of fidelity with respect to the observed data;
3. capable of efficiently estimating rapid workload variations and sudden/unpredictable changes.

## Related work

In this section, we review existing works relevant to workload modeling in order to set the perspective for our contributions. The present review emphasizes, but is not limited to, virtualized cloud environments, such as Google real traces and Open IMS datasets. This overview aims to present a broader picture of general types of workloads and workload modeling techniques that have been previously addressed by the research community.

### Workload domains

Workload domain characterization (i.e.: an heterogeneous cloud infrastructure, a network-heavy IMS telecom infrastructure, a cluster of Web servers, etc.) is the first element that should be considered when planning performance evaluation. It has a major impact on the type of workload to be considered. Domains vary in shape and scope, depending on the size and purpose of the environment; one can go from the performance evaluation of a single application on a workstation, to a full-scale performance evaluation of a multi-tenant, heterogeneous cloud environment (e.g., Amazon EC2). Google clusters and virtualized IMS cloud environments, both subjects of this work, are considered as workload domains belonging to the field of cloud computing. Different relevant works have been cited in this context.

Moreno et al. [3] provide a reusable approach for characterizing cloud workloads through large-scale analysis of real-world cloud data and tracelogs from Google clusters. In the present work, we address workload estimation for the same workload domain. There are, however, differences in their approach, starting with the dynamic behavior of their workload, in contrast with the static behavior of ours. For instance, the evaluation of static versus dynamic workloads will depend on the objective to be achieved. Since jobs from a static workload are processed by a “clean” system, there is usually an implied need to evaluate a system’s behavior over a short time span. Jobs processed in a dynamic manner, on the other hand, require the evaluation of a system’s behavior over a longer time span (several hours, days), since the same job sets are being processed by a system that is continually processing other jobs.

Static workload evaluation [4, 5] usually involves the analysis of a smaller number of workload types and attributes, which increases the complexity of the process. Since job execution times are not considered, this evaluation incurs a “drift” in variation in the rate of usage resources, since the system keeps processing jobs stacked in its queue while new jobs arrive. Dynamic workload evaluation such as in [1, 6] overcomes this issue by adding a probabilistic and/or distributed (e.g., normal distribution, exponential distribution) approach to job arrivals and execution times, which provides a more accurate representation of the impact of workload types and attributes on the system resources.

MapReduce and Hadoop performance optimization [3–5] is also an interesting avenue for study because of the nature of the data-intensive computing taking place in such environments, and because this framework is at the core of most of the leading tech company datacenters worldwide, such as Google, Yahoo and Facebook. This type of optimization, on the other hand, focuses on long-term analysis of predictable workloads and scheduled tasks, rather than on quick bursts in demand for a specific application in a non-predictable manner. The performance evaluation of Web and cloud applications ([1, 5, 6]) is another popular domain worthy of consideration. Among other things, this domain usually involves the evaluation of user behavior, which is less prevalent in other domains. However, the domain is typically application-centric and rarely considers the workload characteristics of the whole cloud environment.

### Workload types

To narrow down our search space further, the next step when planning performance evaluation is to define the workload types to investigate. Feitelson [2] gives a good description of the constitution of a workload type. For instance, the basic level of detail of a workload is considered an instruction, and many of them compose a process or a task. A set of processes or tasks can in turn be initiated by a job sent by a user, an application, the operating system, or a mix thereof.

In the field of cloud computing, performance evaluation aims mainly to optimize hardware resource utilization. Workloads typically originate from a mix of user, application and task workload types. For instance, the workloads studied by Magalhaes et al. [1] and Bahga et al. [6] are spawned from the behavioral patterns of specific user profiles using selected Web applications. A similar work by Moreno et al. [3] uses a similar approach, but focuses on the workload processed within a cloud datacenter driven by users and tasks. Studying these workload types can be useful in evaluating our own datasets, since the latter also depend on user behavior. In fact, user behavior is the prime factor in workload generation for our performance evaluations. Another approach proposed by An et al. [5] consists in viewing the user, the application and the service workload types as three layers of granularity. The users launch a varying amount of application-layer jobs, which in turn execute a varying number of service-layer tasks. This method is effective in the context of predictable, dynamic workloads. In some cases, only one workload type will be investigated, such as parallel processes and jobs under a specific cloud environment [4].

To summarize, work in this context aims to observe very specific benchmarks from selected applications. In the case of this paper, for instance, we have specifically chosen workload traces from the telecom, web and cloud domains because the workload types greatly vary from one another. Telecom workload types rely on SIP register and invite requests to establish a media session throughout a service function chain. This implies that each VM running specific functions, jobs and tasks are more homogeneously distributed (core infrastructure, edge infrastructure, user devices) across the infrastructure than those of a public cloud infrastructure such as a Google datacenter.

### Workload attributes

Finally, consideration of workload attributes is the last step when planning the performance evaluation of a system. Attributes are what characterize workload types, and directly influence the system’s hardware resources. For instance, I/O (disk and memory usage, network communications, etc.) attributes include the distribution of I/O sizes, file access patterns and the use of read and/or write operations [2].

In the present work, only the scheduling of the CPU is of interest. Hence, the relevant attributes are each job’s arrival and running times [2]. CPU scheduling is a common interest in performance evaluation, and most studies analyze many workload attributes related to this resource. In An et al.’s work [5], the system CPU rate, threads, Java Virtual Machine (JVM) memory usage and system memory usage are analyzed. Similar attributes are considered in Magalhaes et al.’s work [1], where the system CPU rate, memory rate and users’ transactions per second are evaluated. Other works are much more thorough in their analysis [3], where attributes are subdivided into user patterns (submission rate, CPU estimation, memory estimation) and task patterns (length, CPU utilization, memory utilization). Setting aside the differences in workload domains, such as IMS and Google performance evaluation, we can safely assume that the system CPU behavior remains the same in both these cloud environments.

### Workload modeling

Workload modeling aims to create workloads that can be used in performance evaluations. Of course, the objective is to get a workload model as close as possible to the real workload [2] It always starts with measured workload data, and is a common alternative to using the traced workload directly to drive simulations.

The most common approach found in the literature to create a workload model is to create a statistical summary of an observed workload. Approaches in [3, 6] amd [1] apply this summarization to all the workload attributes (e.g., CPU, memory usage, I/O, etc.), and then fit distributions to the observed values of the different parameters. In one case, for example, a statistical analysis of user requests is performed to identify the right distributions that can be used to model the workload attributes such as the inter-session interval, the think time and the session length [6]. Four candidate distributions are then considered for each workload attribute based on efficiency: exponential distribution for inter-session arrival, hyper-exponential distribution for modeling think times and inter-session intervals, and Weibull distribution and Pareto distribution to model session lengths.

Some approaches use more sophisticated techniques for online prediction. For instance, the work in [12] proposes an online incremental learning approach to predict the runtime of tasks in scientific workflows in clouds. Their approach harnesses fine-grained resources monitoring data in order to improve the performance of the predictions. This technique is particularly useful in real-time, non predictable workload environments since it has the ability to capture sudden changes in the cloud’s performance. Next, the approach in [13] proposes an online method that first estimates task runtime, disk space and peak memory consumption based on the size of the task’s input data. It then looks for correlations between the parameters of a dataset to generate task estimates to finally estimate workflow task requirements based on a MAPE-K (Monitoring, Analysis, Planning, Execution, Knowledge) loop. While these techniques are efficient at predicting online task runtime and requirements, they do not solve the problem of generating “normal” and continuous usage of system resources in order to create generic load profiles. These load profiles are particularly useful for Cloud Service Providers (CSPs) and Telecom Service Providers (TSPs) alike to anticipate resource provisioning demand in their respective environment.

Other approaches applied to entirely different fields can also be of great interest. For instance, Tahmasbi and Hashemi [17] propose a model for forecasting urban traffic volume by using, among other things, the Hull-White model. This model is almost exclusively used in the finance sector to predict fluctuations in stock prices over a short period of time. In the case of short-term urban volume, the Hull-White model has provided interesting results, and we expect it will do the same for our needs. Also noteworthy is the use of quadratic splines with irregular intervals as statistical summaries, and the use of the Weiner process as a distribution fit.

Other existing works have proposed workload prediction using linear regression [18], Neural Networks [19], a Kalman filter [20, 21] and SVR [22]. An optimal strategy for resource management should allow dynamic on-demand adjustment and provisioning of resources. This can be greatly facilitated if the workload can be predicted accurately. In this context, Hu et al. [23] proposed to address CPU usage estimation based on time series. Their approach is based on support vector regression and Kalman filter, particularly smooth Kalman filter, in order to remove the noise in the data, and reduce the prediction error rate. Higher weights are assigned to the latest data in the training set as such data provide more recent information on the behavior of the system. This idea was developed previously by Cao et al. [24] in their study of SVR with adaptive parameters in the prediction of time series in the financial domain. These approaches have outperformed prediction algorithms which are based on linear regression and neural networks, and even SVR.

## Methodology

In this section, we present an overview of the proposed scheme for our proposed approaches as well as a thorough description of the numerous algorithms and equations involved.

### Workload generator architecture

**Fig. 1:**
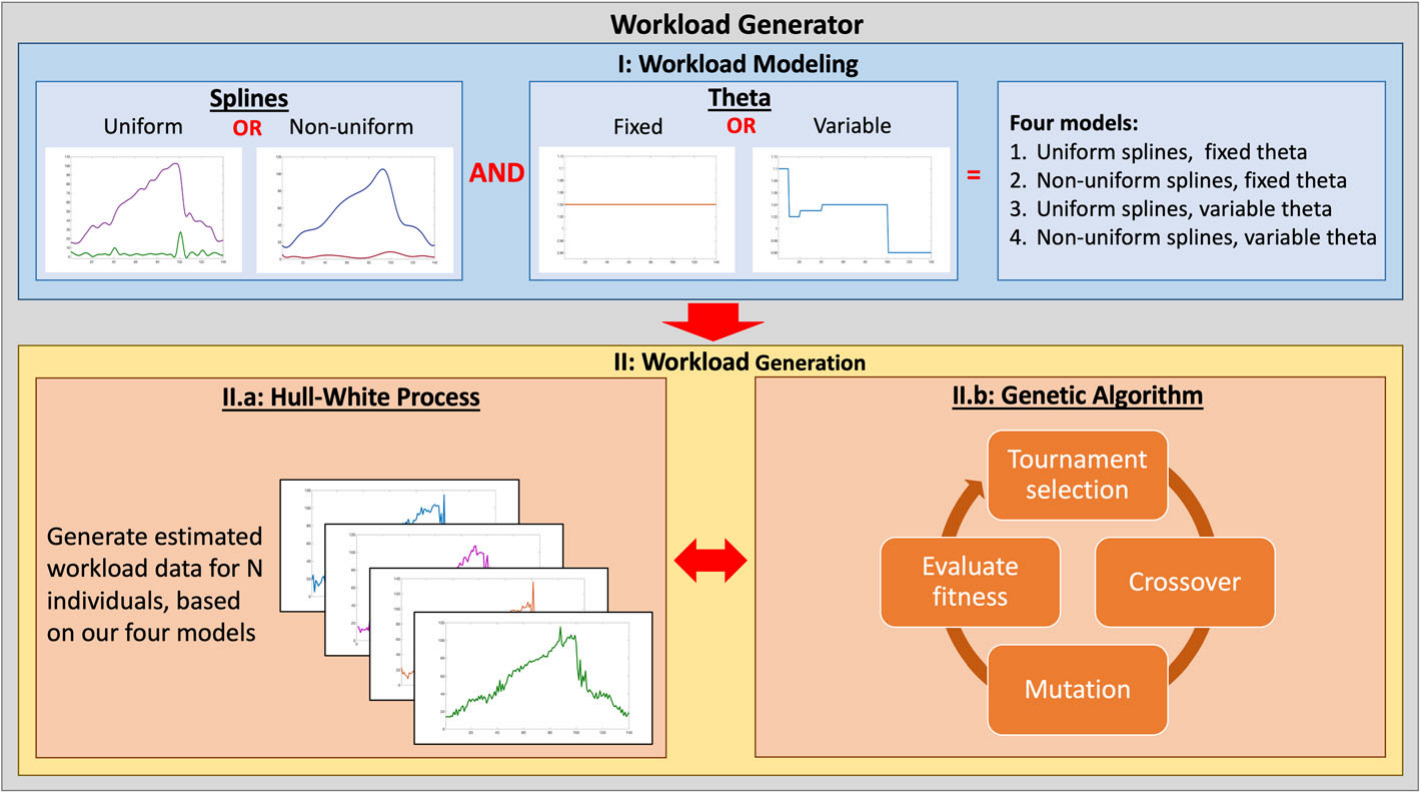
Proposed Workload Generator Scheme

For the sake of clarity, we summarize the different steps involved in the Hull-White GA workload generator in Fig. 1, which can be used as reference. The novelty of our approach lies in the relationship between (1) the Hull-White workload modeling process and (2) a custom GA involved in a workload generation and optimization process. For Hull-White modeling, we first estimate the mean and the standard deviation of an observed workload data set in chronological order. To get smooth and continuous mean and standard deviation functions, our workload generator employs uniform and non-uniform quadratic spline curves. Next, fixed and variable *θ* values (*θ*, a Hull-White parameter) will be estimated. The full Hull-White modeling process is explained in sub-section III.B. The breakdown of segments for non-uniform splines, as well as for variable *θ* values, are evaluated in an entirely automated process by the workload generator. The latter sets boundaries in which large variations exist in the values of the mean and/or standard deviation over a short period of time. We thus obtain four different Hull-White models from which to generate our workload data, namely: (1) uniform splines and fixed *θ*, (2) uniform splines and variable *θ*, (3) non-uniform splines and fixed *θ* and (4) non-uniform splines with variable *θ*.

On its own, the Hull-White modeling process proves to be an efficient algorithm for estimating workload data. However, by enhancing the Hull-White modeling with a custom GA optimization, our experiments demonstrate that the MAPE of the estimated data generated by our approach is significantly improved, with minimal impact on the execution time. That proves to be especially true when pairing the benefits of the GA with the four aforementioned Hull-White models, each providing different levels of fidelity, and excelling in different areas.

To briefly summarize the steps needed to obtain estimated workload data through this workload modeling approach: (1) we provide discrete observed workload data as an input to the workload generator, (2) this workload data is processed and four Hull-White workload models are automatically generated and saved under a workload profile, (3) a workload profile is used in all or in a part of a workload generation process, and (4) the custom GA generates estimated workload data from many instances of the corresponding workload profile(s) and proposes the fittest solutions.

### Hull-white workload modeling

In this sub-section, we present the formulation of the workload modeling problem and its underlying processes. The main objective of this process is to develop realistic workload profiles for different virtualized telecom and IT systems, based on data obtained from real systems, without requiring knowledge of their inner working processes (Black-box approach).

#### Stochastic differential equations (SDEs)

From each consecutive sample of the real data, we generate mean and standard deviation values. SDEs constitute an excellent choice to model the time evolution of dynamic systems subject to random changes.

1 $$ d{X}_t=\mu (t) dt+\sigma (t)d{W}_t,\kern2.5em t\ge 0 $$

where:

*X*_*t*_ : Observed workload.

*μ*(*t*): Mean value of observed workload at time t.

*σ*(*t*): Variance value of observed workload at time t.

*W*_*t*_ : Weiner process.

#### Splines

**Fig. 2:**
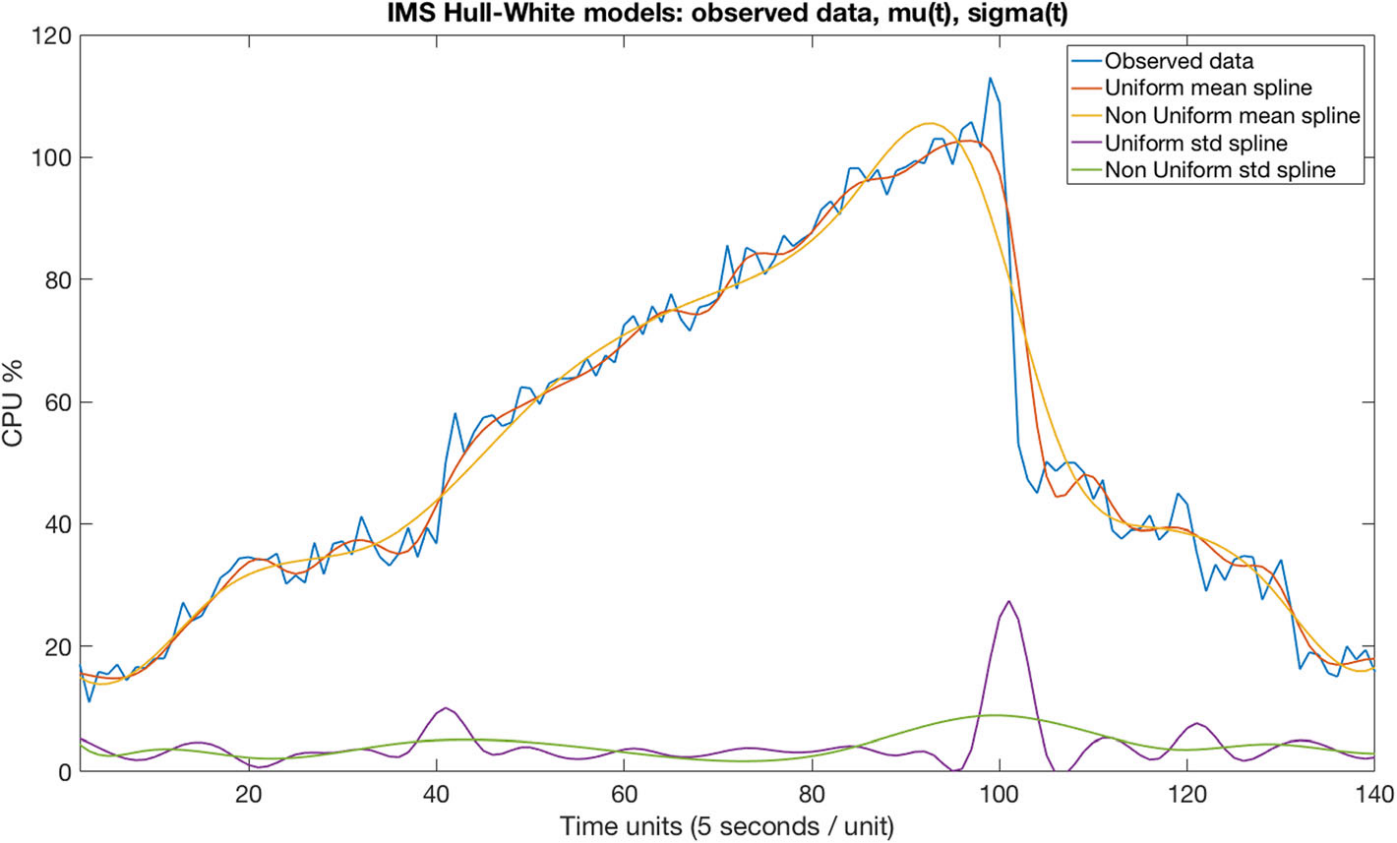
IMS1 Hull-White sampled splines: Observed data, μ(t), σ(t)

Next, we generate splines for curve-fitting continuous mean *μ(t)* and continuous standard deviation *σ(t)* values as outlined in Fig. 2. Splines are used to estimate the mean *μ(t)* and standard deviation *σ(t)* of a set of workload data in order to achieve smooth and continuous functions. We use both uniform and non-uniform splines. The first one uses knots (aka “anchor points”) set at regular time intervals (e.g., one knot every 20 s), while non-uniform splines use knots set at irregular time intervals.

2 $$ \hat{f}\left({X}_t(t)\right)=\left\{\begin{array}{c}{a}_i{t}_{i-1}^2+{b}_i{t}_{i-1}+{c}_{i-1}=\hat{f}\left({X}_{i-1}\right)\\ {}{a}_i{t}_i^2+{b}_i{t}_i+{c}_i=\hat{f}\left({X}_i\right)\kern4em \\ {}{a}_i{t}_{i+1}^2+{b}_i{t}_{i+1}+{c}_{i+1}=0\kern3em \end{array}\right.\kern0.75em i=1,\dots, n $$

where:

*a*_1_ = 0 : (first linear spline).

$$ \hat{f}\left({X}_t(t)\right)=\mu (t) $$ : Continuous mean.

or

$\hat{f}\left({X}_t(t)\right)=\sigma (t)$ : Continuous standard deviation.

#### Hull-white process

The following describes the main properties of the Hull-White model, which is a popular SDE choice in the finance sector for modeling future interest rates. Note that Fig. 2 depicts how uniform and non-uniform splines are generated from the sample of an observed IMS CPU workload, thus providing *μ*(*t*) and *σ*(*t*) for our Hull-White models.

3 $$ d{X}_t=\left(\mu (t)-\theta {X}_t\right) dt+\sigma (t)d{W}_t,\kern2.5em t\ge 0 $$

where:

*X*_*t*_ : Observed workload.

*μ*(*t*): Mean spline value of observed workload at time t.

*σ*(*t*): Standard deviation spline value of observed workload at time t.

*θ* : Estimated parameter.

*W*_*t*_ : Weiner process.

#### Estimation of θ

The parameter *θ* is a key feature of the Hull-White process. It is an estimated value that provides a drift (an upwards/downwards motion) to the estimated workload. In the present work, we generate models with both fixed and variable *θ* values. For fixed *θ*, we take the whole data of size *T* and calculate one *θ* value for the dataset. As for variable *θ*, we use time windows whose size *T* depend on variations in the data.

4 $$ {L}_t\left(\theta \right)=\exp \left(-\underset{0}{\overset{T}{\int }}\hat{u}\left(t,{X}_T\right)d{X}_T-\frac{1}{2}\underset{0}{\overset{T}{\int }}{\hat{u}}^2\left(t,{X}_T\right) dt\right) $$

where:

5 $$ \hat{u}\left(t,{X}_T\right)=\frac{\mu (t)-\theta {X}_T}{\sigma (t)} $$

Therefore, the Maximum Likelihood Estimation (MLE) is defined as

6 $$ \hat{\theta_T}=\arg \mathit{\max}\ {L}_T\left(\theta \right) $$

### GA workload estimation

A GA is a metaheuristic inspired by the natural selection process. It belongs to the larger class of evolutionary algorithms (EAs), and is commonly used in computer science to generate optimized solutions to complex search problems. To this end, a GA relies on bio-inspired operators such as mutation, crossover and selection to simulate the propagation of the fittest individuals over consecutive generations.

#### Individuals

In GA, a population is a set of *n* individuals that form potential solutions. For workload estimation, we define each individual/chromosome as a set of decimal values. Each chromosome is an estimated workload value for the current workload attribute of the workload profile under evaluation. For instance, if we evaluate the CPU attributes of an IMS workload profile, each individual would be composed of chronologically and randomly estimated CPU workload values provided by any of the defined Hull-White models.

#### Segments

An individual is divided into smaller segments, or genes, to allow it to proceed further in an evolution process of crossover and mutation operations. Each segment is of varying size and represents a portion of the individual. For instance, an individual of length L = 100 can be divided into 4 even parts, hence creating 4 segments, with segment 1 containing genes 1 through 25; segment 2, genes 26 through 50, etc.

#### Fitness function

In GA, a fitness function is required to rank individuals in the current population. The score of an individual depends on how close its estimated values are to the observed values of a workload profile. In the problem at hand, we evaluate the fitness of an individual by its MAPE score:

7 $$ MAPE=\frac{100}{n}{\sum}_{t=1}^n\left|\frac{X_t-{\hat{X}}_t}{X_t}\right|\kern0.5em $$

where:

*X*_*t*_ : Observed value.

$$ {\hat{X}}_t $$ : Estimated value.

#### Selection

During each successive generation, individuals of the current population are selected to breed a new population (hence the term “generation”). For the purpose of our workload-modeling problem, we proceed by tournament selection. To this end, we pick one candidate with the fittest solution amongst x randomly selected individuals. The process is then repeated to select a second candidate. Both candidates become parents to generate a new offspring in the crossover operator.

#### Crossover operator

The crossover operator generates a next-generation population of solutions from those chosen through tournament selection. Segments are chosen randomly, based on a uniform rate of *ρ*_*c*_ (probability of selecting segments from one parent over the other), from both parents *I*_1_ and *I*_2_ to generate a new offspring.

#### Mutation operator

The mutation operator generates new estimated values from a randomly selected Hull-White model for randomly selected segments of an individual. It is based on a mutation rate (probability of mutating a segment) of *ρ*_*m*_.

#### Algorithm

Algorithm 1 describes the proposed process for workload modeling with GA. It starts by generating a random population “P” of “*N* = 52” candidate workload values, or individuals (Line 4), and then evaluates the fitness function, being the MAPE for each individual (Line 5 to 11). Next, a range of “T = 5” random candidates is selected among population P, and the candidate with the best MAPE is chosen through a tournament selection process (Lines 15 and 16). Afterwards, the two newly selected candidates, or parents, go into the evolution crossover process, with the offspring being a random composition of candidate1’s genes (*ρc*) and candidate2’s genes (1- *ρc*), with *ρc = 0.5* (Line 17), to generate a new individual, or offspring for the next generation. After a new population of “N” individuals is.

generated in this manner, the whole population goes through a mutation process (Line 20). This mutation process evaluates a randomly generated number, for each segment of each individual, with a mutation rate “*ρm = 0.015*”. If the segment’s rate is lower than.

the mutation rate, it is replaced by a new segment generated with one of the randomly selected Hull-White models. Next, the fitness of these individuals forming the new population is calculated (Line 22 to 28). The process is repeated until the maximum number of generations “*G* = 60” is reached. Finally, the algorithm returns the fittest individual having the lowest MAPE found in the last generation (Line 31).

### SVR-Kalman workload estimation

Beside the Hull-White-GA, we also propose and study a Kalman filter [8] and SVR [9] combination for workload estimation. The Kalman filter is a well-known model for estimating a hidden state x of the system indirectly from measured data, and it can integrate data from as many measurements as are available [8]. The Kalman filter model is defined as follows:

8 $$ {x}_k={Ax}_{k-1}+{Bu}_{k-1}+{w}_{k-1} $$

With a measurement *z*,

9 $$ {z}_k={Hx}_k+{v}_k $$

where:

*A* : Transition matrix from time *k* − 1 to *k*.

*B* : Control matrix

*u*_*k* − 1_ : Known vector

*H* : Matrix showing the relationship between *z*_*k*_ and *x*_*k*_

*w*_*k* − 1_ and *v*_*k*_ : Process and measurement noise, respectively

After filtering observed workloads with the Kalman filter to remove the noise and hence minimize the prediction error, we use SVR to estimate workloads. In SVR, input data are separated into training data classes using linear hyperplanes. If they cannot be linearly separated, then input vectors (observed data) are mapped onto a high-dimensional feature space using a non-linear mapping function (kernel function). SVR identifies the optimal hyperplane that maximizes the margin between the vectors of the considered classes. This optimal hyperplane is defined as a linear decision function (find optimal parameters *w* and *b*):

10 $$ f(x)= wK(x)+b $$

where:

*x* : Input data, *w* is the weight vector and *b* is the bias parameter

*K*(*x*) : Kernel function (e.g., linear, Radial Basis Function (RBF))

Algorithm 2 shows the proposed Expectation Maximization Algorithm (EM) EM-KalmanFilter-SVR. The algorithm starts by initializing SVR parameters (Line 1). The Kernel function model can be Radial Basis Function (RBF), linear, or polynomial. The parameter C trades off misclassification of training examples against simplicity of the decision surface, while Gamma can be defined as the inverse of the radius of influence of samples selected by the model as support vectors [25]. Then, another initialization phase is conducted for the data samples of the Kalman filter. Next, the initial state mean is set to 0 while n_dim_obs, the size of the observation space, is set to 1 (Line 3). The estimated state is calculated using EM (Lines 4 and 5). EM is a meta-algorithm for learning parameters in probabilistic models. It aims to find parameters that maximize the expected likelihood of the observed variables. The EM algorithm is used in the present work to estimate model parameters with the Kalman filter. The algorithm estimates the state with the Kalman filter (Line 4), and then estimates it with filtering and smoothing (Line 5). Afterwards, both the estimated state and the smoothed state are used in SVR for filter prediction and smooth filter prediction, respectively (Lines 7 and 8). Finally, the algorithm returns the filtered estimated data and the smooth filtered estimated data (Line 9).

## Experimental results

In this section, we present the results of our experiments.

### Use cases

In our experiments, we used CPU workload from two different domains: IT and telecom. Another scenario use telecom throughput workload to validate the genericity of our approaches. We selected these workloads because they differ significantly from each other, and we wanted to evaluate the performance of our approach under different load behaviors while also assessing its general efficiency under various situations. Hence, to cover as many scenarios as possible, we used 5 different datasets: 2 CPU workload scenarios and 1 throughput scenario from the telecom domain (IMS1, IMS2, ThrpIMS) with a similar configuration, but with slight variations in the amount of customer calls per second (CPS) generated, and 2 from the IT domain (Google, BitBrains). The Google dataset was taken from a single server in a Google cloud environment, and showed a standard CPU workload behavior under normal utilization in a cloud environment. The BitBrains dataset was a business-critical CPU workload trace from a single VM, collected from a distributed cloud hosting datacenter graciously provided by BitBrains IT Services Inc. These traces are freely available through the public Grid Workloads Archive [26].^1^ Lastly, the last dataset (ThrpIMS) is incoming throughput workload traveling through a virtual Interrogating Call Session/Control Function (I-CSCF) server from a virtualized IMS infrastructure.

### Configuration

To demonstrate the proposed hybrid workload modeling approach, we created workload models based on observed CPU workloads of virtualized IMS, Google and BitBrains clusters as well as throughput from a virtualized IMS infrastructure. The workload modeling experiments were performed on a server with a 2.6 GHz Intel Core i7 6-core processor, 16 GB of RAM, and using Matlab R2018a. The following describes the configurations and scenarios used in this paper.

#### IMS1 configuration

The IMS1 dataset is a collection of CPU workload data obtained by stressing an IMS virtualized server with call setups. Table 1 describes the amount of calls generated in a given time frame, for the first cycle.

**Table 1:** IMS1, Cycle 1 Configuration

Phase	Starting CPS	Variation	Duration
1	150	+ 50 CPS/10 s.	50 s.
2	400	–	100 s.
3	600	–	300 s.
4	200	−50 CPS/50 s.	150 s.

The next 5 cycles are variations of the first cycle, as shown in Table 2, with different CPS rates and durations.

**Table 2:** IMS1, Configuration Variations, Cycles 2–6

Cycle	Variation from 1st cycle
2	+ 50 CPS
3	−50 CPS
4	−100 CPS
5	−25 CPS
6	+ 300 CPS

#### IMS2 configuration

The IMS2 dataset is another collection of CPU workload data obtained by stressing an IMS virtualized server with call setups. Table 3 describes the amount of calls generated in a given time length, for the first cycle.

**Table 3:** IMS2, Cycle 1 Configuration

Phase	Starting CPS	Variation	Duration
1	150	+ 50 CPS/50 s.	150 s.
2	500	+ 50 CPS/50 s.	100 s.
3	900	+ 50 CPS/50 s.	150 s.

The next 5 cycles are variations of the first cycle, as shown in Table 4, with different CPS rates and durations.

**Table 4:** IMS2, Configuration Variations, Cycles 2–6

Cycle	Variation from 1st cycle
2	−100 CPS
3	+ 275 CPS
4	−25 CPS
5	+ 400 CPS
6	+ 25 CPS

#### Google configuration

CPU load collected every 300 s of a single machine in a cluster captured and made available by Google.

#### BitBrains configuration

CPU load collected every 300 milliseconds from a virtual machine hosting business-critical applications.

#### ThrpIMS configuration

Incoming throughput, in megabytes per second (Mbps) collected every 5 s from a virtual I-CSCF.

### Results

In the first set of experiments, we have generated workload data from a single Hull-White model combined with GA as follows:

- Model 1: uniform *μ*(*t*) and *σ*(*t*) splines, fixed *θ*
- Model 2: non-uniform *μ*(*t*) and *σ*(*t*) splines, fixed *θ*
- Model 3: uniform *μ*(*t*) and *σ*(*t*) splines, variable *θ*
- Model 4: non-uniform *μ*(*t*) and *σ*(*t*) splines, variable *θ*

In the second set of experiments, we combined each of the four Hull-White models with GA, with the former used to model workloads and the latter to generate optimized workload estimates. Further, we compared all these models with SVR and SVR-Kalman models. SVR allows data approximation based on statistical learning theory. In this SVR model, the prediction of future resource usage was based on observed data, which we divided into training and prediction sets to generate the estimated workloads. As for the SVR-Kalman model, we filtered observed data through the Kalman filter, and then we made predictions using SVR. For SVR, we used four observations to train the model and two observations to estimate the next two values. The kernel function was set to RBF since it is more appropriate to use on nonlinear datasets, while C and Gamma values were fixed at 0.1. These parameters were set through extensive tests performed in order to find the configuration minimizing the estimation error. As for the Kalman filter, we considered the transition as an identity matrix, we assumed a vector of zero control input, and the noise measurement was of the state directly. Therefore, we set *A* = 1, *u* = 0, and *H* = 1 in Eqs. 8 and 9, which we define as follows:

11 $$ {x}_k={x}_{k-1}+{w}_{k-1} $$

12 $$ {z}_k={x}_k+{v}_k $$

**Fig. 3:**
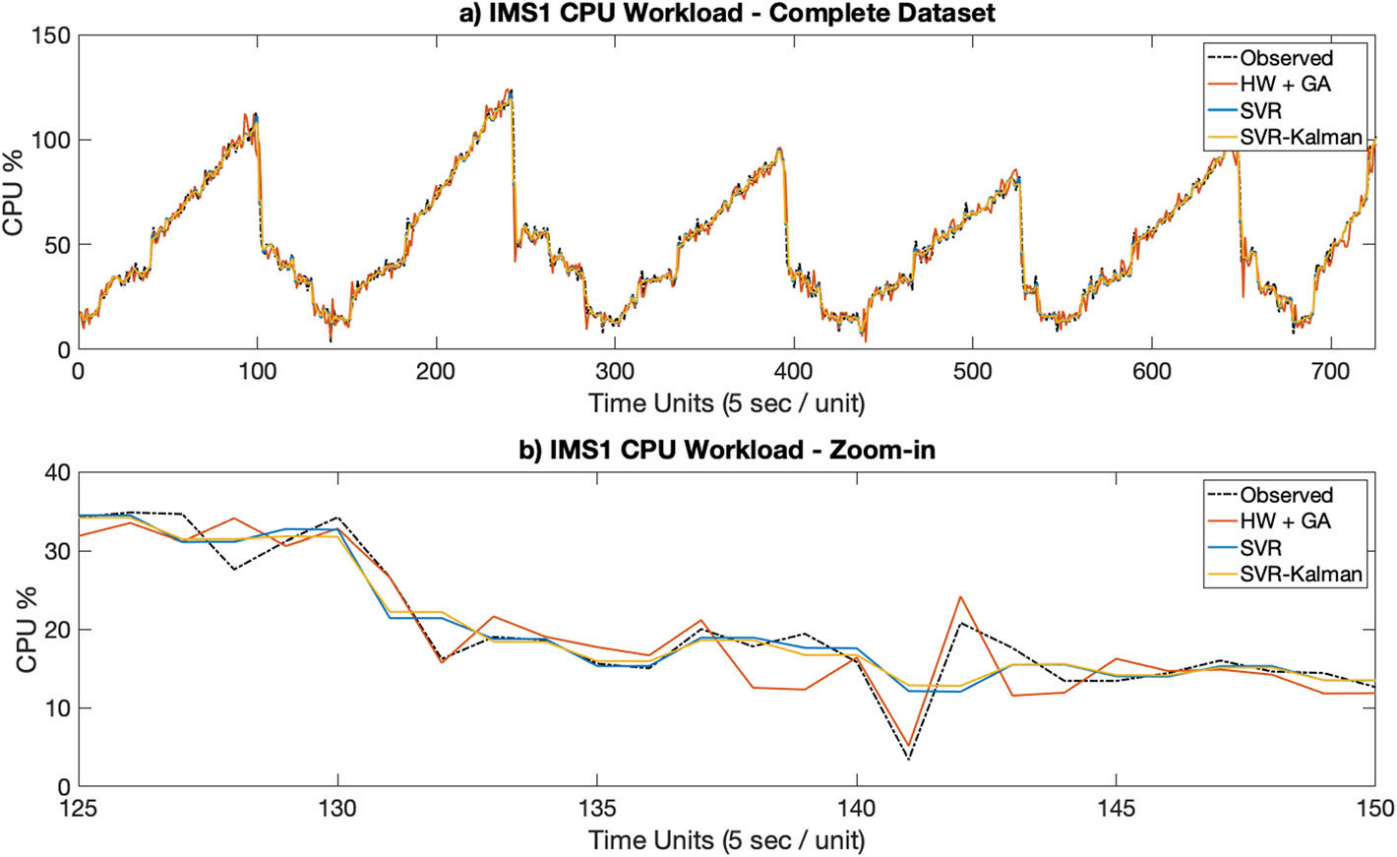
IMS1 Observed and Estimated data: **a** Complete dataset, **b** Zoom-in

**Fig. 4:**
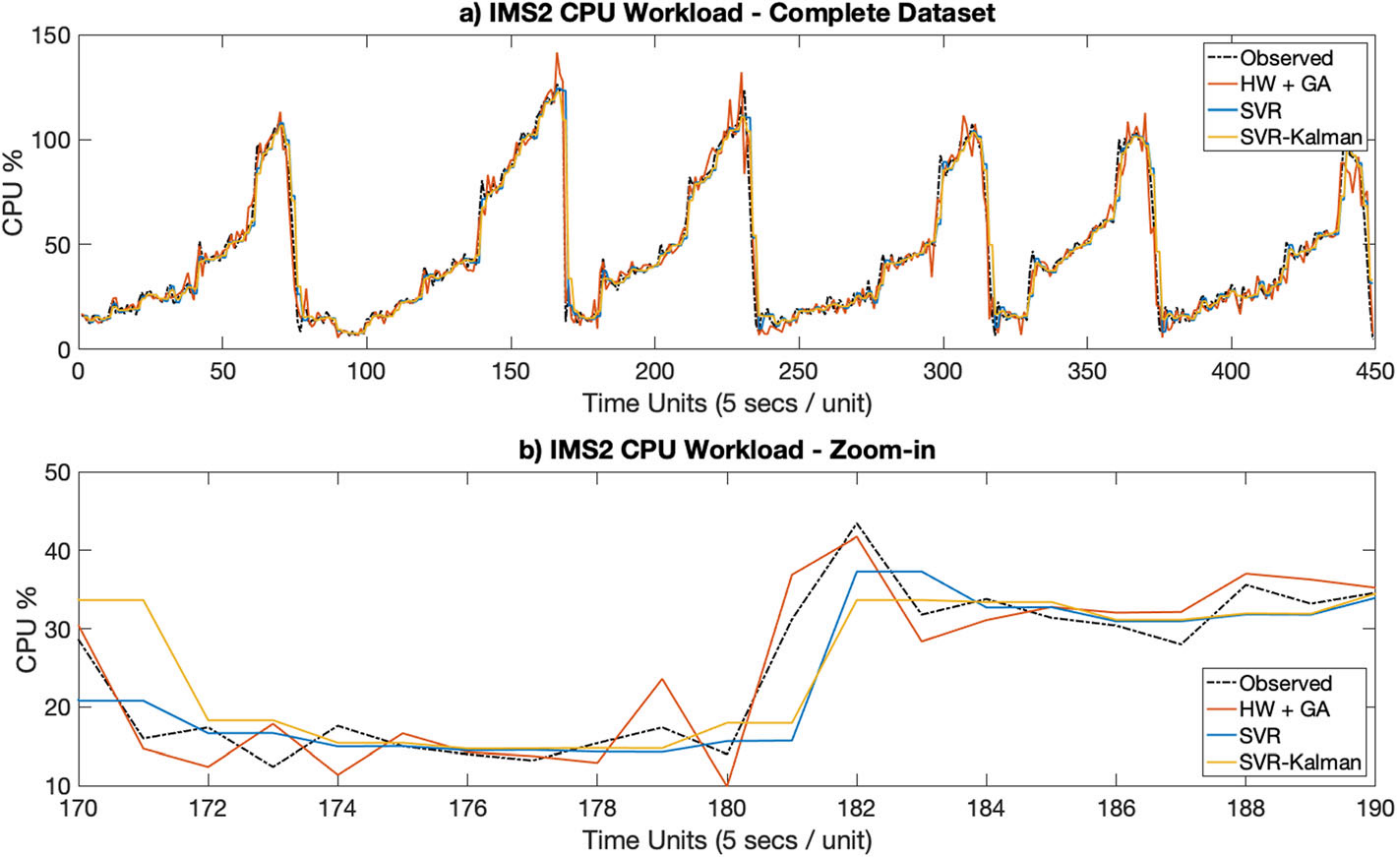
IMS2 Observed and Estimated Data: **a** Complete dataset, **b** Zoom-in

**Fig. 5:**
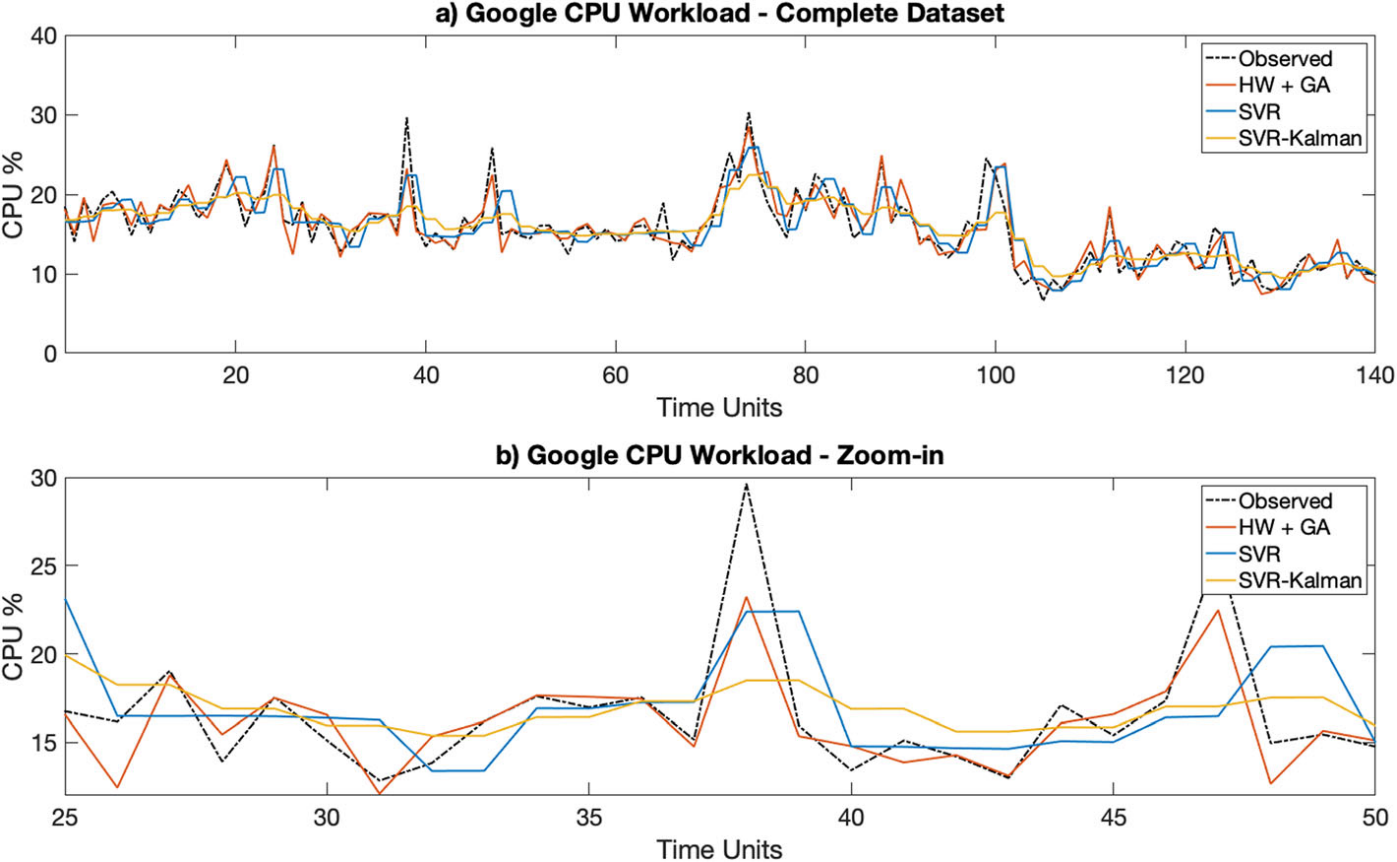
Google Observed and Estimated data: **a** Complete dataset, **b** Zoom-in

Figures 3a 4, 5, 6, 7a depict the results of the Hull-White-GA, SVR and SVR-Kalman estimated workloads for each dataset, while Figs. 3b, 4, 5. 6, 7b show zoomed-in workload estimations of each dataset.

**Fig. 6:**
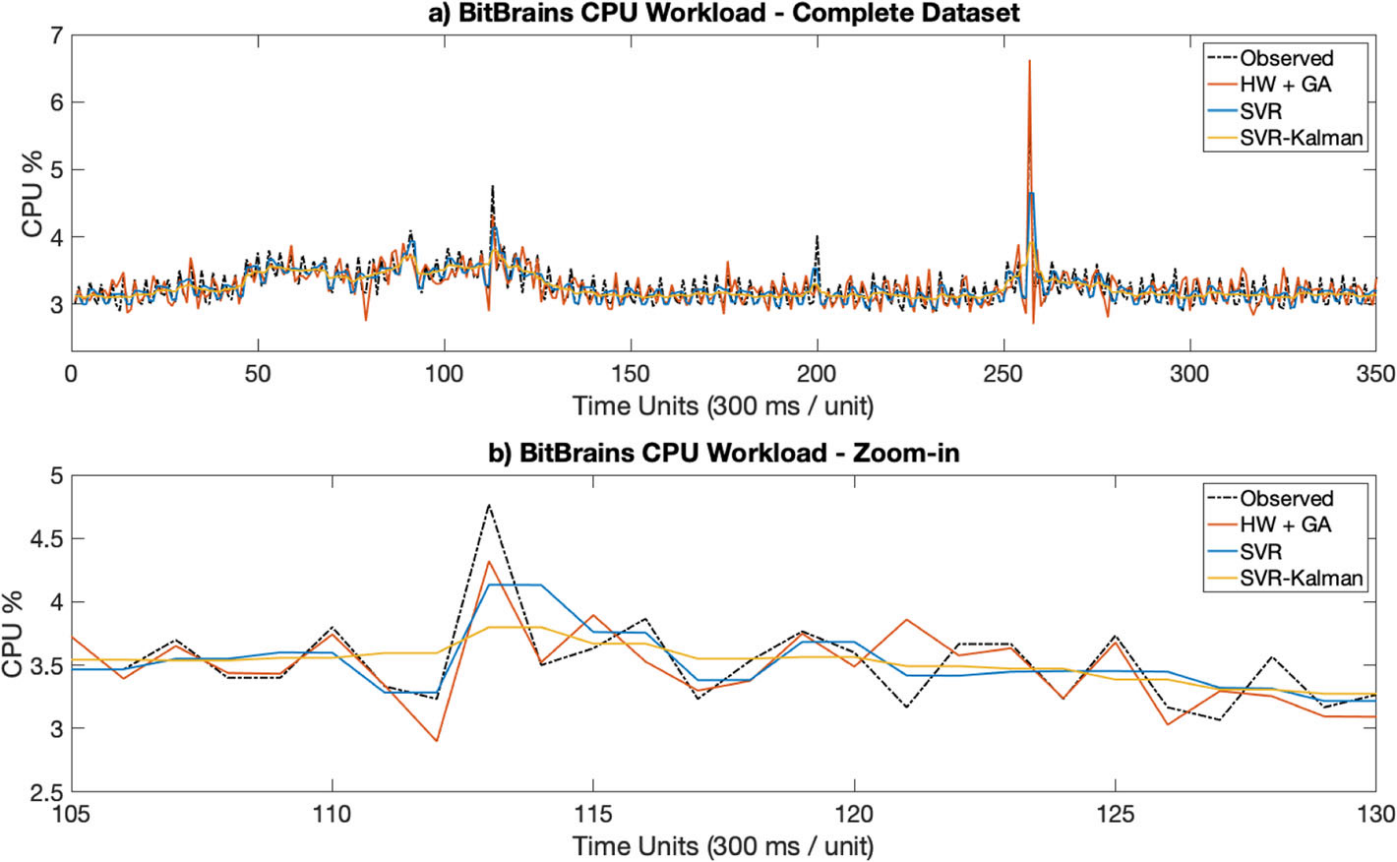
BitBrains Observed and Estimated data: **a** Complete dataset, **b** Zoom-in

**Fig. 7:**
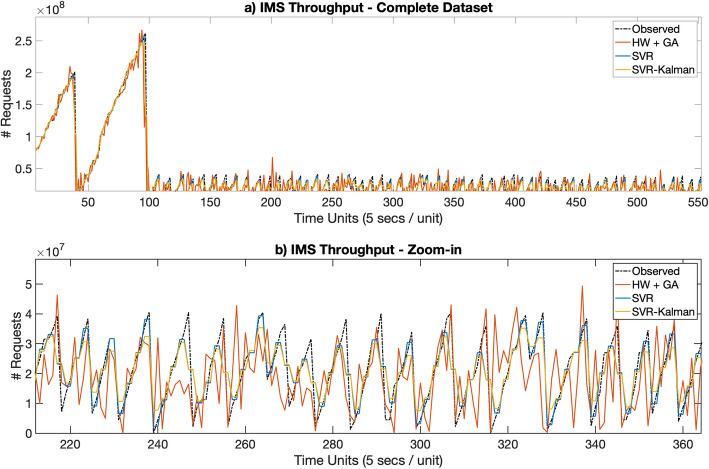
IMS Throughput Observed and Estimated data: **a** Complete dataset, **b** Zoom-in

In Figs. 3 and 4, results show that the Hull-White-GA telecom estimated CPU workloads faithfully match the

observed CPU workloads’ behavior and that the amplitude in estimated workload variations, enhanced by a drift effect from the *θ* values from the Hull-White models, provide more realistic, unsteady workload fluctuations. SVR and SVR-Kalman estimated workloads, on the other hand, show steadier workload fluctuations which is helpful in predicting future.

CPU wokload usage, but doesn’t quite represent the nature of the workloads’ behavior.

Similar observations can be made from IT CPU workloads depicted in Figs. 5 and 6, with the difference that areas with sharper variations in the observed workload cause the standard deviation of the Hull-White models to increase significantly, thus generating estimated workload data with larger variance in some intervals. While these areas still show a similar behavior to the observed workload, the overall accuracy of the Hull-White-GA approach tends to diminish by a small margin.

Figure 7 shows steadier telecom throughput observed workload. In this case, SVR and more particularly SVR-Kalman outmatch Hull-White-GA. In this scenario, the drift effect incurred by Hull-White’s *θ* values hinders the Hull-White-GA approach by infusing unwanted, unsteady throughput variations in the estimated workload.

Next, Table 5 gives the average standard deviation of the MAPE of the estimated workload, based on all Hull-White and Hull-White-GA scenarios, and finally, Table 6 gives the average execution time of each approach, based on 10 simulations. In Table 6, we observe that the execution times of the SVR and SVR-Kalman approaches are significantly faster than that of Hull-White-GA. This is explained by the fact that both SVR and SVR-Kalman are machine learning-based approaches based on statistical learning theory. They estimate workload based on past workload usage in a short time window. Hull-White-GA, on the other hand, applies a segmentation process of the entire dataset and runs through multiple generations to generate potential candidates. This consumes a lot of processing power which lengthens considerably the runtime of the workload generation process. We may also add that the runtime for Hull-White models 1, 2, 3 and 4 are not listed since they get marginally better results than those based on the full GA-Hull-White approach. This is explainable due to the fact that all of these approaches get through the same GA process with the same number of iterations and all other hyperparameters (tournament selection, mutation rate, etc.) are the same.

**Table 5:** Average std. of MAPE based on 10 simulations

Dataset	GA-Hull White	H-W Model 1	H-W Model 2	H-W Model 3	H-W Model 4
IMS1	0.204	0.839	0.808	0.529	0.784
IMS2	0.284	1.593	0.897	0.995	1.050
Google	0.317	1.036	0.982	0.875	1.377
BitBrains	0.071	0.289	0.262	0.281	0.374
ThrpIMS	1.847	158.737	1.290	1.655	1.457

**Table 6:** Average execution time (seconds) based on 10 simulations, 60 generations for GA

Dataset	GA-Hull White	SVR	SVR-Kalman
IMS1	6.15	0.0481	0.0464
IMS2	4.03	0.0286	0.0278
Google	2.32	0.0094	0.0091
BitBrains	3.84	0.0228	0.0219
ThrpIMS	6.89	0.060.8	0.0603

Lastly, Table 7 illustrates the MAPE of the considered models. The best result for each scenario is depicted in bold. In Table 7, for instance, we observe that MAPE from Hull-White-GA is generally much better than those of each individual Hull-White model 1–4 but also very close to the SVR and SVR-Kalman results based on telecom (better) and IT (worse) CPU workloads. In scenarios involving telecom throughput, however, SVR-Kalman significantly outmatches other approaches.

**Table 7:** Average MAPE of solutions based on 10 simulations

Dataset	GA-Hull White	Hull-White Model 1	Hull-White Model 2	Hull-White Model 3	Hull-White Model 4	SVR	SVR-Kalman	Num samples
IMS 1	**6.668**	12.734	15.728	12.352	14.641	14.30	10.72	724
IMS 2	**9.300**	21.903	27.345	21.736	26.196	26.02	16.66	448
Google	7.275	18.454	20.715	17.713	21.105	19.19	**5.82**	138
BitBrains	3.7024	7.378	8.197	7.472	7.737	6.78	**1.31**	351
ThrpIMS	63.864	650.605	70.031	62.016	72.416	165.70	**0.68**	550

The results show that Hull-White combined with GA maintains a MAPE in the 5 to 10% range for both telecom and IT CPU estimated workloads, while also maintaining by far the highest fidelity to the observed workload for all tested datasets. Moreover, Figs. 3b, 4b, 5b, 6b and 7b show that the Hull-White-GA estimated workload fits the observed workload, with the exception of some areas where there are sharp CPU variations. It is not the case, however, for SVR and SVR-Kalman estimates, following a flatter pattern which does not faithfully represent the observed workload behavior.

To summarize, these experiments show that Hull-White combined with GA is able to provide high accuracy for workload modeling and estimation, with the highest fidelity to the workload behavior, but with higher overhead in terms of execution time as compared to SVR-Kalman. Yet, the latter loses efficiency proportionally to the segment size, and is more sensitive to large variations and peaks in the observed workload data. Overall, both approaches provide distinct advantages and tradeoffs: Hull-White-GA has a much higher runtime and is only effective in offline environment, for cases when we look for estimated data with the highest fidelity to the observed workload. SVR-Kalman, on the other hand, outperforms Hull-White-GA and is a good candidate for online predicgtion. Its fidelity to the observed workload is, however, extremely low.

## Conclusion and future work

For dynamic on-demand adjustment and provisioning of resource needs in the cloud environment, an accurate prediction of the system behavior is needed. The assessment of system behavior requires large amounts of workload data. To address the need for real workload data, something that is hard to obtain, we proposed in this article two novel paradigms for workload emulation, namely, a Hull-White model combined with a custom GA and a support vector regression model optimized with a Kalman filter. We evaluated both techniques on different datasets of IMS, Google and BitBrains CPU and throughput workloads. The results show the advantage of the Hull-White GA model over SVR and SVR-Kalman, manifesting higher fidelity for IMS, Google and BitBrains CPU and throughput datasets. As for the Google and BitBrains CPU as well as the IMS throughput workload data, SVR-Kalman shows better results in terms of the least MAPE. However, for all datasets, SVR-Kalman outperformed both the SVR and Hull-White GA with negligible execution time, showing that this approach is a good candidate for online prediction. Such promising results pave the way for a valuable track to examine the proposed hybrid workload modeling approaches on other workload attributes, such as RAM, disk I/O and network traffic.

## Data Availability

Data is available upon request to the corresponding author.

## Abbreviations

Cloud: cloud computing
CSP: Cloud service provider
CPU: Central processing unit
DL: Deep learning
EA: Evolutionary algorithm
EM: Expectation maximization algorithm
GA: Genetic algorithm
IMS: IP Multimedia subsystem
IT: Information technologies
I/O: Input and output
JVM: Java virtual machine
MAPE: Mean absolute percentage error
NFVI: Network function virtualization infrastructure
QoS: Quality of service
RAM: Random access memory
RBF: Radial basis function
SDE: Stochastic differential equation
SLA: Service level agreement
SLO: Service level ojective
SVR: Support vector regression
Telecom: telecommunications
TSP: Telecom service provider
VM: Virtual machine
VMM: Virtual machine monitor

## Footnotes

1 These traces can be accessed at http://gwa.ewi.tudelft.nl/datasets/BitBrains
